# Visual Function Improvement after Plasma Exchange Therapy for Acute Optic Neuritis in Neuromyelitis Optica Spectrum Disorders: Case Series and Review

**DOI:** 10.3390/diagnostics14090863

**Published:** 2024-04-23

**Authors:** Raluca Iancu, Ruxandra Pirvulescu, Nicoleta Anton, George Iancu, Sinziana Istrate, Mihaela Oana Romanitan, Aida Geamanu, Matei Popa Cherecheanu

**Affiliations:** 1 Department of Ophthalmology, “Carol Davila” University of Medicine and Pharmacy, 050474 Bucharest, Romania; raluca.iancu@umfcd.ro (R.I.); sanzinici@yahoo.com (S.I.); aida.geamanu@umfcd.ro (A.G.); 2 Department of Ophthalmology, “Grigore T. Popa” University of Medicine and Pharmacy, 700115 Iasi, Romania; 3 Department of Obstetrics-Gynecology, “Carol Davila” University of Medicine and Pharmacy, 020956 Bucharest, Romania; klee_ro@yahoo.com; 4 Department of Internal Medicine, Section of Neurology, Södersjukhuset, 11883 Stockholm, Sweden; mihaela.romanitan@regionstockholm.se; 5 Department of Cardiovascular Surgery, “Carol Davila” University of Medicine and Pharmacy, 050474 Bucharest, Romania; matei_cherecheanu@yahoo.com

**Keywords:** optic neuritis, neuromyelitis optica spectrum disorder, visual function, plasma exchange therapy

## Abstract

Objective: Neuromyelitis optica (NMO) and neuromyelitis optica spectrum disorder (NMOSD) are autoimmune-mediated central nervous system disorders distinguished by the presence of serum aquaporine-4 IgG antibody (AQP4-Ab). The clinical panel comprises severe optic neuritis (ON) and transverse myelitis, which can result in incomplete recovery and a high risk of recurrence. Methods: This study aimed to evaluate the visual outcomes of three patients with severe acute ON in NMOSD that was non-responsive to intravenous methylprednisolone (IVMP), who received plasma exchange therapy (PLEX). We included three patients (P1, P2 and P3) with severe acute ON who had no improvement after IVMP treatment and were admitted to the ophthalmology department at the Emergency University Hospital Bucharest from January 2022 to September 2023. All three patients with ON were diagnosed in accordance with the criteria described by the Optic Neuritis Treatment Trial. All the subjects were experiencing their first attack. Results: The mean recruitment age was 35.3 ± 7.71. All patients were seropositive for the AQP4 antibody. All patients were tested for serum myelin oligodendrocyte glycoprotein (MOG) antibody but only one showed a positive test (P3). Lesions visible in orbital MRI indicated the involvement of retrobulbar, canalicular and/or intracranial segments. All three subjects had no response or incomplete remission after an IVMP protocol (5 days of 1000 mg intravenous methylprednisolone in sodium chloride 0.9%). The mean time from onset of optic neuritis to PLEX was 37.6 days. The PLEX treatment protocol comprised five cycles of plasma exchange treatment over 10 days, with a plasma exchange session every other day. An amount of 1 to 1.5 volumes of circulating plasma were dialyzed for 2–4 h. At 1 month after the completion of PLEX therapy, BCVA and VF parameters were improved in all three patients. Conclusion: The treatment of ON remains subject to debate and is somewhat controversial. Plasma exchange must be considered as a rescue therapy when IVMP is insufficient for AQP4-ON patients. This study revealed that PLEX treatment effectively improves the visual outcomes of patients experiencing their first attack of severe acute isolated ON after high-dose IVMP treatment. This study suggests that PLEX may be associated with improved visual outcomes in NMOSD acute optic neuritis.

## 1. Introduction

Optic neuritis (ON) is an inflammatory optic neuropathy affecting one or both optic nerves that can be caused by multiple factors [1,2]. Despite the fact that this entity is classically divided into “typical” or “atypical” entities, there is no consensus regarding the classification of optic neuritis and precise diagnostic criteria are not available [3].

The epidemiology of ON has been studied less carefully than the epidemiology of multiple sclerosis (MS) [4]. Cohort studies with more than 11 million patients report an ON incidence of 3.74 per 100,000 person-years, affecting 114 people per 100,000 of the population in 2018 [4]. Typical optic neuritis, predominantly associated with multiple sclerosis (demyelinating ON), represents the most common type of the disease. The classical triad of symptoms for typical ON comprises vision loss (variable), periocular pain and dyschromatopsia. The prognosis is generally favorable, with good visual recovery regardless of treatment [5]. In contrast, atypical ON has inconsistent visual recovery. Atypical ON can have a wide range of etiologies, like neuromyelitis optica (NMO) and neuromyelitis optica spectrum disorder (NMOSD), autoimmune optic neuropathy, optic neuropathies from systemic disease, chronic relapsing inflammatory optic neuropathy, myelin oligodendrocyte glycoprotein (MOG) and idiopathic neuroretinitis [6]. The typical and atypical ON entities have differences in clinical presentation, biomarkers, imaging findings, response to treatment and visual outcomes [7,8]. Accurate diagnosis of ON at presentation can facilitate the timely treatment of individuals with MS-, NMOSD- or MOG-antibody-associated disease [3]. If left untreated, atypical ON can lead to devastating visual results; therefore, it is crucial to recognize it, initiate proper treatment and preserve vision for patients with atypical ON.

NMO and NMOSD are autoimmune-mediated central nervous system disorders distinguished by the presence of serum aquaporine-4 IgG antibody (AQP4-Ab) [9]. The clinical panel comprises severe optic neuritis and transverse myelitis, which can result in incomplete recovery and a high risk of recurrence [10]. Until two decades ago, NMO was considered a subtype of MS due to their similar clinical presentations. However, in 2015, after the disclosure of the association between AQP4-Ab and NMOSD [11], Wingerchuk et al. proposed new diagnostic criteria for NMOSD, namely, the International Consensus Diagnostic Criteria [12]. Since NMO/NMOSD is a rare and severe disease, there is limited evidence for current guidelines for therapeutic management [13]. Most treatment recommendations are based on case reports, case series and retrospective or prospective studies. It is recognized that intravenous methylprednisolone (IVMP) could improve visual acuity and preserve retinal nerve fiber layer thickness in optic neuritis associated with NMOSD [14,15]; however, some patients have no improvement after receiving IVMP as the first treatment [16]. Several studies have confirmed the efficacy of plasma exchange therapy (PLEX) as a rescue therapy to corticosteroid-resistant optic neuritis, specifically in patients with NMOSD [17,18,19,20].

This study aimed to evaluate the visual outcomes of three patients with severe acute ON in NMO that was non-responsive to intravenous methylprednisolone (IVMP), who received plasma exchange therapy (PLEX).

## 2. Methods

In this retrospective case series, we included 3 patients with severe acute ON who had no improvement after IVMP treatment and were admitted to the ophthalmology department at the Emergency University Hospital Bucharest from January 2022 to September 2023. Written informed consent was obtained from all three subjects. All three patients with ON were diagnosed in accordance with the criteria described by the Optic Neuritis Treatment Trial [21]. The following criteria were used: (1) the presenting episode was the first attack of acute visual loss in one or both eyes, accompanied or not by ocular pain; (2) no presentation of neurological disability other than visual impairment at the onset; (3) severe visual loss described as best-corrected visual acuity (BCVA) less than 20/200 at the onset; (4) visual field loss correspondent with the symptomatology; (5) presence of relative afferent pupillary defect; (6) presence of optic disc oedema [22]; (7) administration of IVMP treatment, 1000 mg/day for 5 days after onset and no other treatment; (8) no visual improvement of more than 2 lines of visual acuity after IVMP treatment; (9) stationary or worsening visual field defects; (10) magnetic resonance imaging performed before IVMP treatment and after PLEX completion. All three cases had irrelevant medical history.

We excluded patients with ON related to MS, patients with optic neuritis treatment already instituted, ischemic optic neuropathy, systemic disease as a predisposing factor for ischemic optic neuropathy, present medication that could possibly give rise to retinal or optic nerve toxicity, traumatic or compressive optic neuropathy and optic neuropathy of an autoimmune or infectious cause.

By reviewing the medical documents, detailed clinical information was collected including age at onset, clinical manifestations, MRI, physical exam, acute phase and maintenance immunosuppressive therapy, PLEX initiation and adverse events associated with PLEX.

## 3. Laboratory Analysis

Epidemiological data show that, cumulatively, ON is most frequently caused by many conditions other than multiple sclerosis [3], so laboratory tests were conducted to exclude the most frequent causes of infectious and post-infectious ON (HbsAg/HIV/HCV/syphilis/toxoplasmosis) or systemic disorders causing ON (thyroid function test, antinuclear antibody test, angiotensin-converting enzyme, mitochondrial DNA mutations, anti-neutrophil cytoplasmic autoantibody, etc.).

The patients’ serum and cerebrospinal fluid (CSF) were collected before PLEX treatment. Complete blood count, fibrinogen levels, prothrombin time, partial thromboplastin time and metabolic spectrum were evaluated before PLEX treatment initiation. The CSF test included total CSF cell counts, white blood cell count, total protein level and glucose level.

## 4. Ophthalmic Examinations

Complete ophthalmic evaluation was performed in all subjects. Evaluations were performed before IVMP treatment initiation, after each day of IVMP therapy, at the end of IVMP treatment and before and after every PLEX therapy session. At each visit, BCVA, pupillary reflex and eye exam, including fundoscopy, were conducted. Before IVMP treatment, at the end of it and before and after each day of the PE treatment visual field exam, contrast sensitivity and color perception were performed and evaluated. The visual field was documented by static automated threshold perimetry using a Humphrey Filed Analyzer II. Color perception was assessed using the Ishihara test and contrast sensitivity via the Pelli–Robson Test.

### 4.1. Neurological Assessment

Neurological exams were performed before and after every type of treatment (IVMP or PLEX therapy). Magnetic resonance imaging (MRI) was performed before IVMP treatment. CSF analysis and AQP4 and myelin oligodendrocyte glycoprotein (MOG) autoantibody serotyping were also performed.

IVMP treatment consisted of 5 days of 1000 mg intravenous methylprednisolone in 0.9% sodium chloride.

### 4.2. PLEX Treatment

Plasmapheresis was considered if optic nerve function had no improvement or incomplete improvement after the IVMP protocol (5 days of 1000 mg intravenous methylprednisolone in 0.9% sodium chloride). With considerations of the possibility of the accumulation of autoantibodies and inflammation, and since this gold-standard treatment (IVMP) was not enough, we considered PLEX treatment, which is a second-line rescue therapy for severe cases resistant to steroids. Standard PLEX therapy consists of five to seven daily sessions every other day requiring close monitoring and management [9]. PLEX treatment was carried on in the resuscitation ward. We decided to include subjects with minimum duration PLEX therapy (5 sessions). PLEX sessions were usually performed in 2 to 4 h, depending on the patient’s height, weight, viscosity of the blood and technical parameters. Daily visual field testing, contrast sensitivity tasting and color perception testing were performed. Plasmapheresis was performed in all three subjects. The treatment protocol comprised five cycles of plasma exchange treatment over 10 days, with a plasma exchange session every other day. An amount of 1 to 1.5 volumes of circulating plasma were dialyzed for 2–4 h. Adverse events associated with the PLEX procedure were recorded, such as nausea, hypocalcemia, hypotension and acute non-occlusive thrombosis.

## 5. Case Presentation

**Figure 1:**
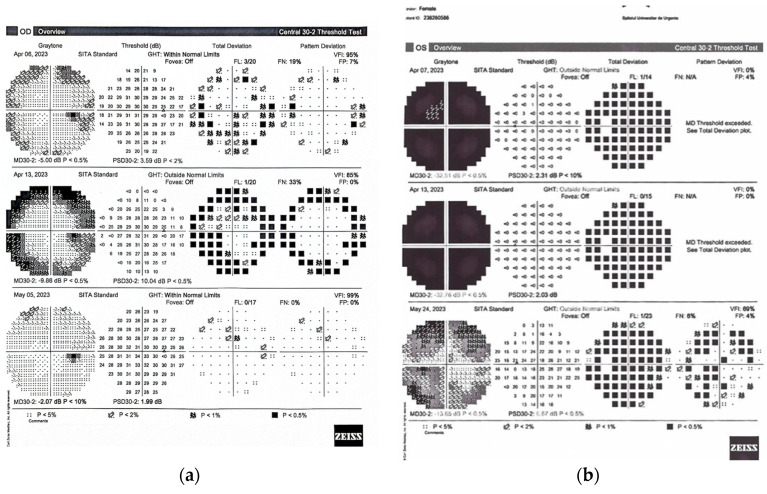
Visual field exams for P1 are presented in (**a**) for the right eye and (**b**) for the left eye. The three sequences correspond to admission, PLEX initiation and 1 month after PLEX.

Patient 1 (P1). A 46-year-old woman was admitted to our clinic with progressive vision loss in her left eye that had begun two weeks earlier; there were significant visual field defects in the left eye at admission. BCVA in right eye was 0.1 logMAR and her left visual acuity was hand movement perception with no improvement after correction and a grade 2 relative afferent pupillary defect (RAPD) in the left eye. Visual field defects were present in both eyes (Figure 1). The eye fundus examination revealed a normal optic disc in her right eye and papillary edema in her left eye. The diagnosis of optic neuritis was considered after MRI showed a slight thickening of the left optic nerve with significant enhancement. IVMP treatment was started immediately after ophthalmic and neurologic evaluation. After 4 days of IVMP treatment, visual field testing showed worsening in her right eye and no improvement in her left eye. PLEX treatment was considered and initiated 7 days from admission. Resolution of ophthalmic symptomatology in the right eye and BCVA and visual field improvement in the left eye appeared at one month after PLEX treatment completion. Visual field exams are presented in Figure 1a for the right eye and Figure 1b for the left eye, at admission, upon PLEX initiation and 1 month after PLEX. Patient characteristics, AQP4-Ab values and ophthalmic assessments prior to and during treatment are provided in Table 1.

**Figure 2:**
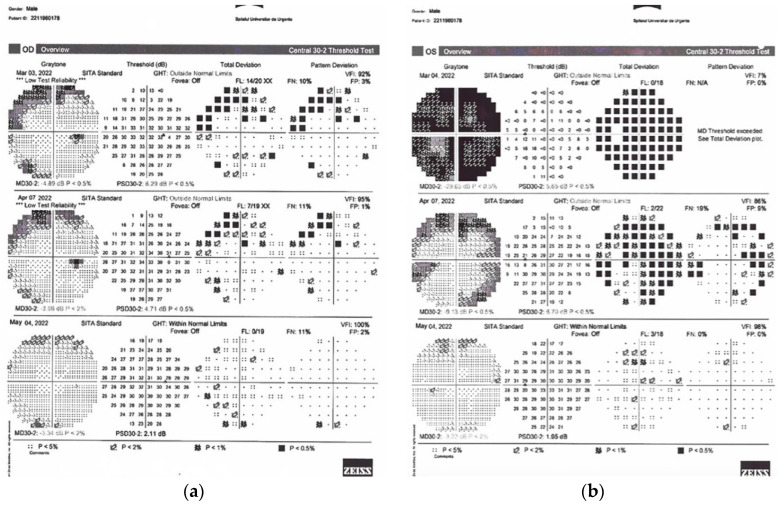
Visual field exams for P2 are presented in (**a**) for the right eye and (**b**) for the left eye. The three sequences correspond to admission, PLEX initiation and 1 month after PLEX.

Patient 2 (P2). A 32-year-old male presented with rapidly progressive visual loss in the left eye with slight pain on extraocular movements for 10 days. His medical history was not significant. BCVA in the right eye was 0.00 logMAR and in the left eye was 1.00 logMAR, with grade 1 RAPD in the left eye. Visual field defects were present in both eyes at admission (Figure 2). The eye fundus exam revealed flu disc margins in the nasal sector of the right eye and papillary edema of the left optic nerve. IVMP treatment was started immediately after ophthalmic and neurologic evaluation. Visual fields improved at one month in both eyes, but significant defects remained and BCVA in the left eye showed no visual improvement of more than 2 lines. PLEX treatment was considered and initiated. Complete ophthalmic improvement appeared after the third PLEX session.

**Figure 3:**
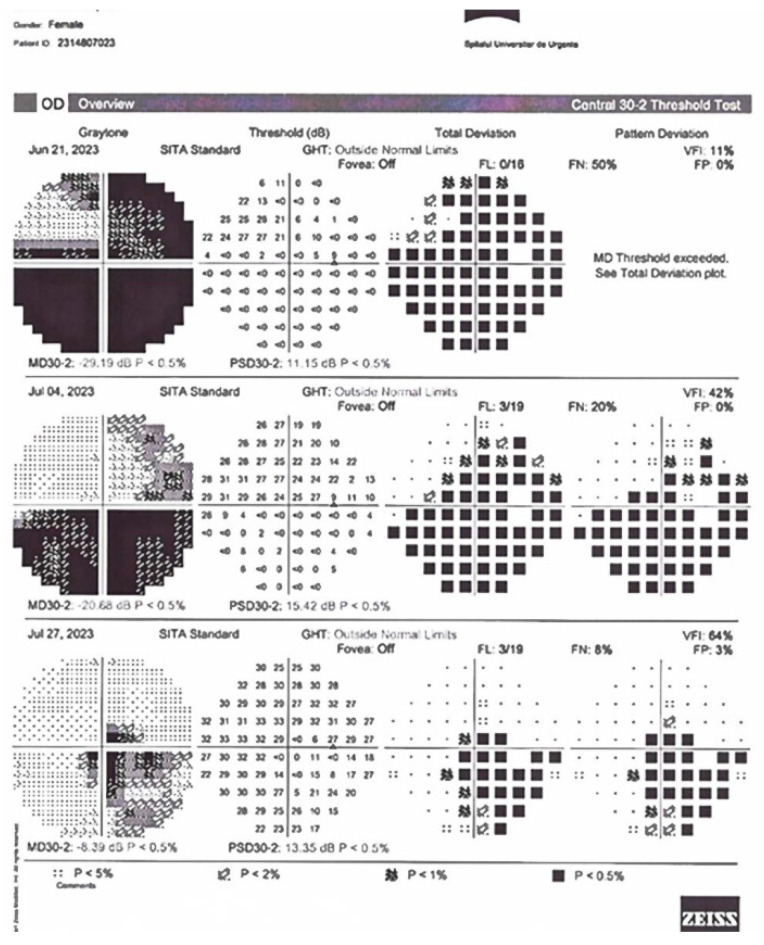
Visual field exams for P3 in the right eye. The three sequences correspond to admission, PLEX initiation and 1 month after PLEX.

Patient 3 (P3). A 28-year-old female was referred to our clinic for the chief complaint of significantly decreased visual acuity in her right eye in the last month. She also had pain with eye movement and headache, with no nausea or vomiting. BCVA in her right eye was 1.3 logMAR and it was 0.00 logMAR in her left eye. Visual field defects and grade 2 RAPD were present only in her right eye (Figure 3). The eye fundus exam revealed significant right optic nerve edema. IVMP treatment was started immediately after ophthalmic and neurologic evaluation, with no significant improvement of BCVA or visual field in the right eye after 3 weeks of corticosteroid therapy. PLEX treatment was considered and initiated. Ophthalmic improvement appeared after completion of PLEX therapy.

## 6. Results

A total of three patients (two female and one male) with severe acute ON in NMOSD were included in this study. Each of the subjects were experiencing their first attack. The mean recruitment age was 35.3 ± 7.71. All patients were seropositive for the AQP4 antibody. All patients were tested for serum myelin oligodendrocyte glycoprotein (MOG) antibody, but only one showed a positive test (P3). Lesions visible in orbital MRI indicated the involvement of retrobulbar, canalicular and intracranial segments. All three subjects had no response or incomplete remission after an IVMP protocol (5 days of 1000 mg intravenous methylprednisolone in 0.9% sodium chloride). The mean time from onset of optic neuritis to PLEX was 37.6 days. The PLEX treatment protocol comprised five cycles of PLEX treatment over 10 days, with a plasma exchange session every other day. An amount of 1 to 1.5 volumes of circulating plasma were dialyzed for 2–4 h. Adverse events associated with the PLEX procedure were recorded, such as nausea (all subjects), low fibrinogen (two subjects), anemia (one subject), hypocalcemia (one subject), hypotension (all subjects) and acute non-occlusive thrombosis (one subject). All recruited subjects completed the full course of five-cycle plasma exchange treatment without any interruption. Early improvement of visual acuity (after the first cycle of PLEX) was found in two out of three subjects (P1 and P2). P3 showed gradual visual improvement from the second to third PLEX cycles. In two patients (P2 and P3), VF MD improved after IVMP therapy but significant defects remained. At 1 month after the completion of PLEX therapy, BCVA and VF parameters were improved in all three patients.

## 7. Discussion

A healthy optic nerve is mandatory for normal visual function. Many conditions can affect the optimal function of the optic nerve, including inflammation, trauma, infection, vascular insufficiency, toxins or tumoral pathologies [23]. Due to the wide range of etiologies associated with ON, the approach to care and treatment may vary depending on the clinical signs, severity of symptoms and etiology [24]. Much of our understanding of the management of ON has been guided by the landmark Optic Neuritis Treatment Trial (ONTT), which demonstrated that high-dose intravenous methylprednisolone led to faster recovery of vision but did not change the outcome for common causes of ON, such as multiple sclerosis-related ON [25,26,27,28]. ON in NMOSD can have devastating visual results if not treated in a timely fashion.

Plasma exchange therapy implies the filtration and replacement of patient’s plasma with artificial plasma [29]. PLEX is considered an alternative treatment for severe demyelinating or antibody-mediated disease. The primary randomized clinical trial demonstrating the efficacy of PLEX was conducted in patients with corticosteroid-resistant demyelinating disease [30]. However, this study did not include ON. Despite the lack of prospective randomized studies on PLEX for ON, there are multiple retrospective studies that have contributed to the increasing utilization of PLEX for ON [31,32]. These retrospective studies have shown that PLEX therapy is associated with better outcomes in NMOSD patients, particularly with early PLEX initiation for those unresponsive to corticosteroids [19,28,31,33,34].

According to the American Society for Apheresis, PLEX is considered as an effective treatment for NMO and NMOSD or multiple sclerosis [35]. PLEX can remove circulating antibodies, complement and cytokines from the blood, which may shorten the action of antibodies and lessen further inflammation and necrosis [36,37], especially in patients with poor response to initial IVMP treatment [38]. The PLEX therapy in this study followed the international standard PLEX protocol, in which 1.5 plasma volumes are exchanged in five cycles over 10 days [39]. PLEX therapy is considered a rescue treatment for ON-NMO resistant to IVMP, and early initiation of PLEX during severe attacks of NMO-SD determines an improved clinical benefit [40].

In our study, we did not limit the time window from onset to PLEX therapy. The time window was under two weeks for one subject (P1). None of our patients had any serious adverse events after PLEX treatment. All subjects included were serum AQP4-antibody positive.

All subjects in our study showed significant visual improvement from the first to the third PLEX cycles. Bonnan et al. also observed significant functional improvement after the second PLEX cycle [41]. A similar result, with significant visual improvement after the second PLEX cycle, was demonstrated by Tan et al. in a study on Chinese patients [22]. Prior IVMP treatment seems also to be a good prognostic factor for visual function improvement [40].

After a literature review was conducted it was observed that are several predicting factors associated with better VA score improvement. One of the most important factors is the time window from onset to PLEX treatment. Earlier treatment of NMO is associated with better outcomes [42]. Tan et al. concluded that a time window of 22.4 ± 11.1 days was significantly related to a better VA outcome [22]. In a recent study, Chen et al. concluded that early PLEX treatment is associated with better outcomes; the median time to PLEX therapy was 2.6 weeks [31]. Fu et al. identified the time window from onset to plasma exchange initiation as the key factor for VA prognosis [43]. The authors calculated a time window as a predictive factor for good outcome that was correlated with VA before PLEX treatment or with the number of ON episodes. The treatment time window was 22 days for patients with HM VA before PLEX treatment and 51.6 days for patients with count fingers (CF) VA before plasma exchange treatment. The recommended time window is within 49.8 days for patients with a first attack of ON, 38.6 days for patients with one previous ON episode, 27.5 days for patients with two episodes, 16.5 days for patients with three episodes and 5.3 days for patients with four episodes [43]. Another study, conducted in Colombia, reports that the time from admission to PLEX initiation and complete improvement at six months was a median of 7 days [37]. Huang et al. concluded in their 2020 study that PLEX can ameliorate severe NMOSD and that PLEX effectiveness was associated with the duration between disease and the initiation of PLEX and the optimal timing for PLEX initiation is 8 to 23 days after the onset of the disease [44]. Early intervention with PLEX should be considered to reduce neurological dysfunction [44]. The number of ON episodes is another important factor for NMO/NMOSD patients. Severe NMO/NMOSD relapses should be considered an emergency and should be treated aggressively from admission [15,18,40,45]. AQP4 seropositivity has been controversial in terms of prognosis. In our study, all subjects were positive for AQP4 antibodies and after PLEX therapy VA logMAR measurements improved markedly after five cycles of plasma exchange treatment. Some articles have reported worse outcomes in AQP4-positive subjects [45,46,47]. The presence of AQP4 IgGs was correlated with attack recurrences [46,48,49] and intrathecal IgG synthesis, lower complement levels and an earlier age of onset [50,51]. Tan et al. revealed that serum AQP4-antibody-positive status had a negative association with better visual outcome [22]. Chan et al. showed that AQP4 + NMO did not have significantly different outcomes from multiple sclerosis and idiopathic optic neuritis in a PLEX cohort, even after controlling for age, gender, severity of vision loss at nadir and time to PLEX treatment [31]. In contrast, other articles have found significant VA improvement after PLEX therapy in AQP4-NO patients [37,43]. Kleiter et al. showed that the serum AQP4 antibody status was associated with better outcomes after PLEX therapy [16,52]. These findings warrant further exploration to determine if AQP4 seropositivity is a positive or negative prognostic factor for VA improvement.

The disadvantages of PLEX are manly related to possible adverse events and the cost of the procedure. In a review published this year, Akosman et al. highlighted the trends in PLEX utilization in ON patients admitted to hospitals in the United States. Compared to ON patients who did not receive PLEX, the PLEX cohort had higher total charges and longer lengths of stay in the hospital [24].

Although PLEX is increasingly used in NMOSD-ON, its therapeutic effect and safety are still controversial [53,54]. The fact that PLEX therapy effectively improves visual outcomes was demonstrated in many recent studies [31,32,33,43,54,55]. Zhang et al. concluded in 2023 that PLEX is an effective and safe therapy for elderly patients with NMOSD and should be considered as a treatment option during NMOSD attacks. In the elderly, preventive measures against hypotension are recommended before PLEX [54]. In this study, functional improvement occurred in 88.0% of the elderly patients 1 month after PLEX and increased to 96.0% after 6 months. This is consistent with another study on NMOSD [56] and supports the long-term benefits of PLEX treatment [57]. There are not sufficient data to produce evidence related to the relapse risk after PLEX therapy; further research is needed in this field. PLEX is an expensive therapy for ON related to NMOSD and serious side effects may occur. Hypotension, risk of infection, hypocalcemia and coagulopathy have been reported; therefore, randomized, prospective studies are still needed. In the subset of ON patients, there was a continuous increase in the utilization of PLEX therapy from 0.63% in 2000 to 2.25% in 2014, and this has increased to an even higher rate year-over-year, from 2.27% in 2016 to 5.56% in 2020 [24].

Alternative immunomodulatory therapies that may offer additional benefit for steroid-resistant acute optic neuritis are represented by intravenous immunoglobulin administration (IVIg) [19]. Recent randomized trials suggest that intravenous immunoglobulin could be a safe and efficacious therapeutic option for the prompt treatment of steroid-resistant acute ON [58,59]. However, the results did not support IVIg therapy alone as a first-line option for acute attacks of NOMSD [59].

This study has some limitations. First, the included patients were all relatively young and all three had improved visual function after PLEX therapy. Second, because IVMP treatments are administered before PLEX is performed in the clinic, the effectiveness of PLEX treatment may be influenced by these medicines.

## 8. Conclusions

The treatment of ON remains subject to debate and is somewhat controversial. Plasma exchange must be considered as a rescue therapy when IVMP is insufficient for AQP4-ON patients. This study revealed that PLEX treatment effectively improves the visual outcomes of patients experiencing their first attack of severe acute isolated ON after high-dose IVMP treatment. This study suggests that PLEX may be associated with improved visual outcomes in NMOSD acute optic neuritis.

## Figures and Tables

**Table 1:** Patient characteristics, AQP4-Ab values and ophthalmic assessments prior to and during treatment.

NO	P1				P2				P3			
Onset age	46				32				28			
Gender	female				male				female			
Affected eye/bilateral	bilateral				bilateral				RE			
Serum AQP4-Ab	1:640				1:320				1:320			
Orbital MRI lesion	Long T2-weighted imaging with enhancement in the posterior 2/3 of the left intraorbital optic nerve; abnormal right optic nerve signal with slight enhancement				Bilateral abnormal optic nerve signal: enhancement in the retrobulbar and intraorbitar segments of left optic nerve; slight enhancement of the right optic nerve				Slight thickening of the right optic nerve with significant enhancement			
BCVA at admission, before IVMP (logMAR)	RE 0.10		LE HM		RE 0.00		LE 1.00		RE 1.30		LE 0.00	
VF at admission (MD30-2/PSD30-2 dB)	RE		LE		RE		LE		RE		LE	
	MD	PSD	MD	PSD	MD	PSD	MD	PSD	MD	PSD	MD	PSD
	−5.00	3.59	−32.8	1.97	−4.89	6.29	−29.6	5.65	−29.1	11.15	−0.29	2.27
Time from onset to PLEX	22 days				47 days				44 days			
Time from admission to PLEX	7 days				34 days				14 days			
BCVA at PLEX initiation	RE 0.3		LE HM		RE 0.0		LE 0.6		RE 1.2		LE 0.00	
VF at PLEX initiation (MD30-2/PSD30-2 dB)	RE		LE		RE		LE		RE		Within normal limits	
	MD	PSD	MD	PSD	MD	PSD	MD	PSD	MD	PSD		
	−12.1	8.55	−32.7	2.03	−3.96	4.71	−9.13	6.70	−20.8	15.42		
BCVA at 1 month after PLEX (logMAR)	RE 0.00		LE 0.10		RE 0.00		LE 0.00		RE 0.50		LE 0.00	
VF at 1 month after PLEX (MD30-2/PSD30-2 dB)	RE		LE		RE		LE		RE		Within normal limits	
	MD	PSD	MD	PSD	MD	PSD	MD	PSD	MD	PSD		
	−2.06	2.08	−13.6	6.67	−3.34	2.11	−3.22	1.95	−8.39	13.35		
OCT at 1 month after PLEX												
Adverse events of PLEX	acute non-occlusive thrombosis hypotension nausea anemia				nausea hypocalcemia hypotension low fibrinogen				nausea hypotension low fibrinogen			

RE: right eye; LE: left eye; HM: hand movement perception; MD: mean deviation; PSD: pattern standard deviation; OCT: optical coherence tomography.
